# Neuroendocrine Liver Metastases and Orthotopic Liver Transplantation: The US Experience

**DOI:** 10.4061/2011/742890

**Published:** 2011-12-29

**Authors:** N. Thao T. Nguyen, Theresa R. Harring, John A. Goss, Christine A. O'Mahony

**Affiliations:** Division of Abdominal Transplantation and Hepatobiliary Surgery, Michael E. DeBakey Department of Surgery, Baylor College of Medicine, 1709 Dryden Road, Suite 1500, Houston, Tx 77030, USA

## Abstract

Liver transplantation remains a controversial therapy for Neuroendocrine liver metastases (NLM), with coflicting suvival data reported. The aim was to assess the evolution of outcomes for patients transplanted for NLM in the US, both before and after the introduction of the MELD scoring system in 2002. The UNOS/OPTN database was reviewed to identify patients diagnosed with NLM who subsequently underwent a liver transplantation from 1988 to March 2011 (*n* = 184); Patient survival was determined using Kaplan-Meier methods and log-rank tests, and cox regression analysis was performed, using SPSS 15.0 (SPSS, Inc, Chicago, IL). The overall NLM patient survivals in the pre-MELD era were 79.5%, 61.4%, and 49.2% at 1, 3, and 5 years, respectively. After the introduction of the MELD score, NET/NLM patients had improved overall patient survivals at 1, 3, and 5 years of 84.7%, 65%, and 57.8%. Patients transplanted after 2002 had an improved survival outcome. Notably, the overall patient survival for NET is not significantly different when compared to the outcomes of patients transplanted for HCC, in the current era. This progress acknowleges the significant improvement in outcomes for NLM patients after liver transplantation and the potential for further gain in the survival of otherwise nonsurgical, terminal patients.

## 1. Introduction

Neuroendocrine tumors (NETs) encompass a broad group of neoplasms which originate from cells of the endocrine and nervous systems and are of similar indolent character. NETs are most commonly located in the gastrointestinal system, including the pancreas, but also arise from many other parts of the body. Patients with gastroenteropancreatic neuroendocrine tumors commonly develop liver metastases, which after protracted periods contribute towards morbidity and mortality [1–4]. In fact, the majority of patients with NET will have liver metastases discovered at the same time as diagnosis [4, 5]. The liver metastases associated with NET are typically multifocal and diffuse, compromising liver anatomy and function. With excess hormone production, these metastases can lead to debilitating symptoms, in addition to end-stage liver disease and death. Patients with neuroendocrine liver metastases (NLM) respond well to surgical resection, but, for patients who are ineligible due to widespread hepatic involvement, orthotopic liver transplantation (OLT) can be considered for curative therapy [4].

NLM is the only acceptable indication for OLT in the setting of metastatic malignancies, enabled by their slow growth rate and relatively low-grade malignancy. However, OLT for NLM remains a controversial therapy as there is conflicting actuarial data comparing outcomes of those transplanted to those who receive other therapies, as well as to others transplanted for different indications. In fact, the difference in 5-year survival between existing patient series can range as wide as 17 to 47%, with one series of 10 patients who received OLT for NET reporting a 90% 5-year survival [2, 6–11]. The lack of consistency in the data is partially due to the rarity of the disease and low incidence leading to small sample sizes. For example, only 14 OLTs were performed for NLM out of 28,665 OLTs performed in the United States in 2010 alone [6]. This is compounded with the wide variety of treatment options and algorithms making standardized and uniform protocols for this patient population difficult.

Surgery is the only potential for cure in NET/NLM patients, currently, and also serves to prolong survival in terminal disease. Complete surgical resection is an excellent therapeutic modality, with acceptable outcomes and minimal morbidity and mortality [12] though unfortunately is available to only 10% of the neuroendocrine cancer patient population [5, 7]. Excessive tumor burden in inaccessible locations precludes complete resection for the majority. Medical treatment options for patients who are not surgical candidates have evolved over the last two decades. This, along with the development of liver-directed therapies including ablative techniques, has expanded the treatment options for the majority of patients with NET/NLM [13].

For those patients in whom surgical resection is not indicated, symptomatic control and improved survival can be obtained with functional hormonal blockade, liver-directed therapies, and aggressive palliative treatments [8, 14]. Otherwise, OLT remains for the patients ineligible for surgical resection or refractory to medical therapies. Currently, patients with favorable biological features, including well-differentiated tumors with low proliferation index and overall stable disease without detectable extrahepatic metastases, may be potentially cured by OLT. Therefore, only a small subset of patients with NLM qualifies for possible OLT. Many transplant programs consider patients with NLM for OLT if several criteria are met: the patient is not a resection candidate, the primary disease is well identified and completely resected, there is no evidence of extrahepatic disease, the patient failed nonoperative therapies, and there is evidence of disease stability for at least a year [9–11, 14]. 

In an effort to assist in the selection of optimal NLM patients who would benefit from OLT, we aim to describe the outcomes of these patients, as well as, explore possible prognostic indicators to improve the allocation of a limited organ supply. Minimal selection criteria exist for this patient population and only recently are prognostic indicators being identified. It is our goal to further characterize this population in an effort to improve the outcomes of those to be transplanted in the future.

## 2. Materials and Methods

The United Network for Organ Transplantation/Organ Procurement and Transplantation Network (UNOS/OPTN) database is a national online database system to collect, store, and publish all OPTN data pertaining to patients waiting for and those who have received transplantation. This system has documented every organ donation and transplantation occurring in the US since 1986 [15]. The UNOS/OPTN database was queried for this study. All OLTs performed between September 1987 and March 2011 were reviewed. Of 108,924 OLTs in the database, 184 were identified to be secondary to neuroendocrine tumors. Since no UNOS/OPTN diagnosis code for neuroendocrine tumor exists, these cases were identified based on the diagnosis text field including “carcinoid”, “glucagonoma”, “gastrinoma”, “insulinoma”, “islet cell tumor”, “pancreatic gastrinoma”, “pancreatic islet cell tumor”, “VIPoma”, “Zollinger-Ellison's syndrome”, “neuroendocrine tumor”, or any combination of those names with “metastatic”, “met”, or “malignant”. The OLTs performed under these classifications are due to an unspecified neuroendocrine liver metastatic disease. Demographic information was analyzed including age, gender, and ethnicity of recipient, along with age of donor. Additionally, recipient characteristics such as creatinine, international normalized ratio (INR), total bilirubin, and albumin at time of transplant, days on the waitlist, model for end-stage liver disease score, or pediatric model for end-stage liver disease score (MELD/PELD), body mass index (BMI), length of hospital stay (LOS) following transplant, and ascites, encephalopathy, or dialysis prior to transplant along with donor characteristics such as creatinine, aspartate aminotransferase (AST), alanine aminotransferase (ALT), and total bilirubin, and operative variables including cold ischemia time (CIT) and warm ischemia time (WIT) were analyzed. The education level of the recipient, whether the recipient received multiple organs at time of transplant, whether the recipient had a prior OLT, or whether the recipient required retransplantation, were also included in the analysis. MELD/PELD scores were introduced in 2002 which changed the paradigm of organ allocation from the Child-Pugh Score and offered a more precise allocation model which accommodates for neoplastic disease. To assess the evolution of OLT outcomes for NLM, the survivals of patients transplanted before and after the introduction of the MELD/PELD system in 2002 were assessed and compared using the Kaplan-Meier and the log-rank tests. Outcomes of all patients transplanted for NET/NLM were then compared to patients transplanted for hepatocellular carcinoma and for nonmalignant indications to assess overall survival experiences of NET/NLM patients relative to other indications.

Time-to-event data were obtained from this database to estimate post-OLT survival. Specifically, the time variable was calculated as the length of time between transplantation and either death or last known follow-up. An observation was censored if the individual was alive at the last known follow-up. Univariate and multivariate analyses were performed by Cox's regression and proportional hazards model, and survival analysis was performed by the log-rank test and the Kaplan-Meier test. Variables in the univariate analysis with *P* value less than or equal to 0.200 were then tested in the multivariate analysis using a step-by-step approach. A *P* value less than or equal to 0.050 was considered statistically significance and indicative of independent prognostic factor. All statistical functions performed on SPSS version 15.0 (IBM SPSS, Chicago, IL, USA).

## 3. Results

### 3.1. Patient Characteristics

**Figure 1:**
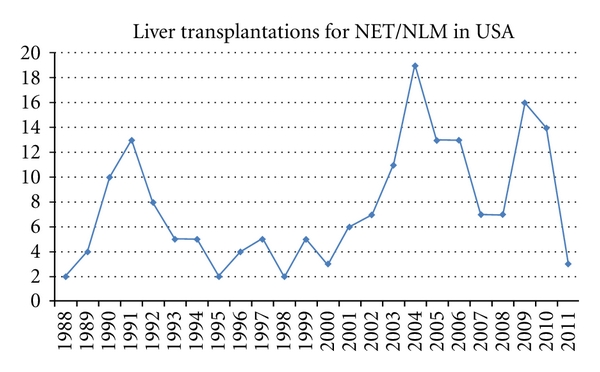
Liver transplantations for NET/NLM in USA. Note the significant increase in average number of transplantation which occurred after the introduction of the MELD/PELD score in 2002.

From 1988 to March of 2011, 108,924 liver transplantations were performed in the US, and, of those, 184 were performed for NET/NLM patients. Descriptive analysis of the sample reveals a slight majority of males at 54.1%, with an average age of 44.9 years (range 11–69 years), a mode of 56 years. Caucasians made up the majority of recipients at 86.5%. The mean survival time was 41 months, though ranged from 0 to 253.3 months at the time this analysis was completed. Average length of stay after transplantation was 22.7 days, ranging 0–5.9 months. The first OLT for NLM included in the UNOS/OPTN database was performed in 1988. The number of OLT performed for the diagnosis of NET/NLM since then is separated into the year when the transplant occurred and is illustrated in Figure 1. Mean wait time on the transplant list for NET patients was 5 months. The larger majority of this subgroup received whole liver allografts, 89.7%, compared to split liver allografts. The LOS after OLT ranged from 15 to 52 days, with a mean of 23 days.

### 3.2. Introduction of the MELD/PELD Score

**Figure 2:**
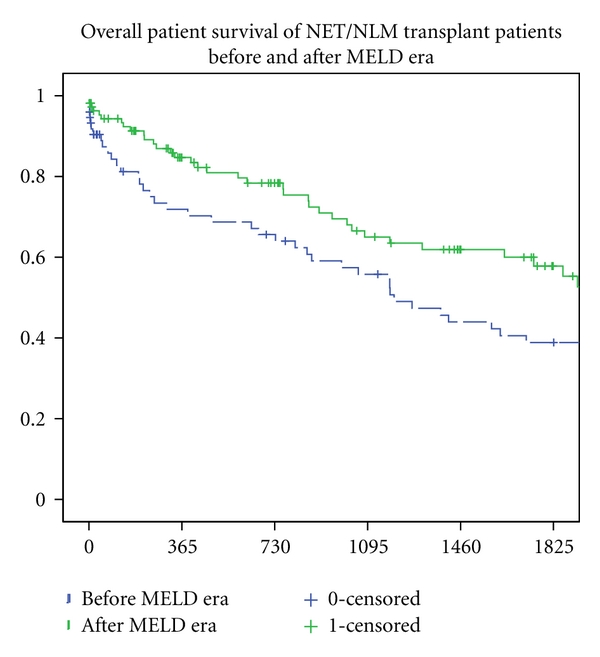
Overall patient survival of NET/NLM transplant patients in pre- and post-MELD era.

Figure 1 illustrates the distribution of liver transplantations which occurred annually since 1988. 74 transplantations occurred prior to the introduction of the MELD/PELD score in 2002. Transplants in the pre-MELD era averaged approximately 4 transplants a year between 1993 and 2001. In the post-MELD era, the average increase to 11.9 transplants annually for NET/NLM. (Please note that at the time this paper was written, 3 transplants occurred as of March of 2011.) The Kaplan-Meier and log rank tests were used to compare the survival experience of both the pre- and post-MELD subgroups and found a statistically significant difference graphed in Figure 2 (*P* = 0.032). Patients transplanted after the introduction of the MELD score had an improved survival outcome as compared to patients transplanted before 2002.

Overview of the patient sample reveals 86 patients who ultimately expired after transplantation for NET/NLM. Forty-six of those were listed to have died from recurrent and metastatic disease. The remaining causes of death are as follows: sepsis/infections (9), unknown (8), lung/kidney/multiorgan failure (7), hemorrhage (2 GI, 2 intracranial), lymphoproliferative disorder (3), graft failure (3), cardiac arrest (2), trauma (1), and hyperkalemia (1).

### 3.3. Patient and Allograft Survival

**Figure 3:**
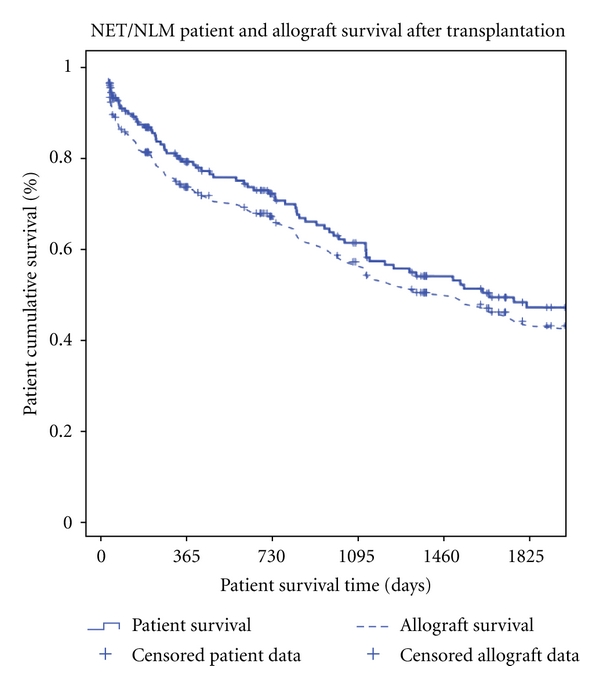
Neuroendocrine liver metastases: patient and allograft survival after transplantation, 1988–2011.

Patient and allograft survival of all NET/NLM patients ranged between 0 and 229.4 months. The mean overall patient survival was 91.9 months (7.5 years) ± 10 months whereas the median overall patient survival was 58.6 months ± 12.8 months. Overall patient survivals were 79.5%, 61.4%, and 49.2% at 1, 3, and 5 years, respectively (Figure 3). Allograft survival was 73.4%, 56.6% and 45.4% at 1-, 3-, and 5-year survivals, respectively, with an overall mean allograft survival of 83.9 months ± 9.3 months and overall median allograft survival of 47.1 months ± 8.2 months (Figure 3). 

**Figure 4:**
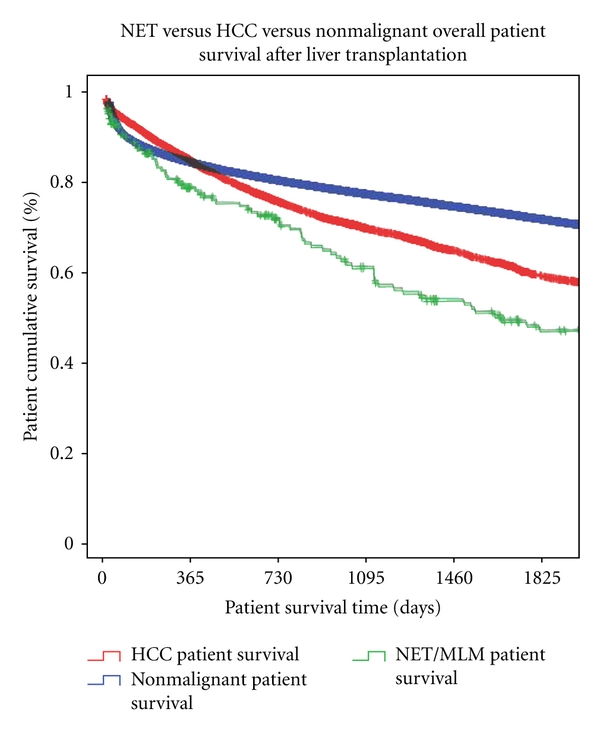
NET/NLM versus HCC versus nonmalignant patient survivals after transplantation, 1988–2011. NET/NLM versus HCC (*P* = 0.002); NET/NLM versus nonmalignant (*P* ≤ 0.00).

Kaplan-Meier log-rank analysis was also applied to overall patient survivals of patients transplanted for hepatocellular Carcinoma and for nonmalignant indications. These survival curves were then compared to all NET/NLM patients since 1988. NET/NLM patients had overall patient survivals of 79.5%, 61.4%, and 49.2% at 1, 3, and 5 years, respectively. This is compared to HCC patients, with 1-, 3-, and 5-year survivals at 85.8%, 71.1%, and 60.6%, significantly lower with a *P* value of 0.002 (Figure 4). Overall patient survival of those transplanted for nonmalignant indications was 85.2%, 78.3%, and 73.0% at 1-, 3-, and 5-year survivals, respectively, significantly better than patients transplanted for NET/NLM (*P* < 0.00) (Figure 4). 

**Figure 5:**
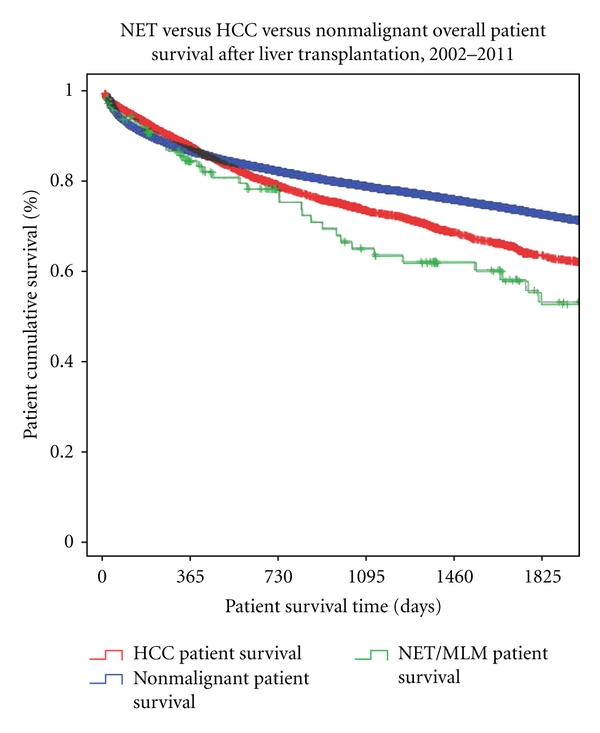
NET/NLM versus HCC versus nonmalignant patient survivals after transplantation, 2002–2011. NET/NLM versus HCC (*P* = 0.109); NET/NLM versus nonmalignant (*P* = 0.002).

In light of the improved survival of NET patients transplanted after 2002, Kaplan-Meier log-rank analysis of overall patient survivals was also done for the three groups (NET/NLM, HCC, nonmalignancy) of patients transplanted after 2002. NET/NLM patients had overall patient survivals at 1, 3, and 5 years of 84.7%, 65%, and 57.8%. This is compared to HCC patients, with 1-, 3-, and 5-year survivals at 88.0%, 74.3%, and 64.4%. As opposed to transplants occurring prior to 2002, this difference in survival between HCC and NET/NLM patients is no longer significant (*P* = 0.109). Overall patient survival of those transplanted for nonmalignant indications was 87.1%, 79.5%, and 73.7% at 1-, 3-, and 5-year survivals, respectively, still significantly better than patients transplanted for NET/NLM (*P* = 0.002) (Figure 5). 

### 3.4. Univariate and Multivariate Analysis of Clinical Variables

Several recipient and donor predictors were analyzed in univariate analysis listed in Table 1. Of these variables, the following approached the *P* value cutoff for significance of <0.20 and were analyzed in the multivariate Cox regression model. Several prognostic factors were statistically significant after multivariate analysis (Table 2).

For all patients transplanted for NLM since 1988, a higher albumin serum level at transplant was significantly protective for patient survival (*P* = 0.033, OR = 0.45). A higher donor creatinine at transplant had a negative impact on allograft survival (*P* = 0.00, OR = 1.32), and, if the patient requires retransplantation, the patient also had worse allograft survival (*P* = 0.004, OR = 49.01; Table 2).

For patients transplanted for NLM *after* January 2002, multivariate analysis revealed that patient survival was deleterious for higher recipient total bilirubin (*P* = 0.02, OR = 1.06) and higher donor creatinine (*P* = 0.004, OR = 1.29) at time of OLT. A higher recipient albumin level at transplant portended a protective effect (*P* = 0.011, OR = 0.48) for patient survival. Allograft survival was negatively impacted if the patient required retransplantation (*P* < 0.001, OR = 35.9) and by higher recipient total bilirubin (*P* = 0.02, OR = 1.06) and higher donor creatinine at time of transplant (*P* = 0.004, OR = 1.31; Table 2).

## 4. Discussion

The use of OLT for NLM remains a controversial topic, in large part because of dismal outcomes, especially in early evaluations of smaller series of patients. Over time, these outcomes have slowly improved in further investigations, with a growing number of larger series done mostly in Europe. In our analysis of the USA experience for OLT due to NLM, we have found that 5-year survival outcomes are less favorable as compared to recipients of OLT for other causes, but in-depth analysis reveals remarkable improvement in the last decade. Of NET/NLM patients transplanted after 2002, with the introduction of the model for end-stage liver disease (MELD)/pediatric end-stage liver disease (PELD) scoring systems for organ allocation [16], the 5-year survival rate increased from 49.2% to 57.8% as compared to all patients transplanted since 1988. Notably, the overall patient survival for NET/NLM is not significantly different from the 1-, 3-, and 5-year outcomes of patients transplanted for HCC in the current era, since the advent of the MELD/PELD scoring system. This improvement acknowledges the potential for further gain in the survival of a patient population that would otherwise be considered for nonsurgical, palliative care. The outcomes are not significantly different from those of HCC patients, in whom liver transplantations are performed regularly. OLT serves as a reasonable effort in a patient group otherwise desperate for aggressive treatment options; the 5-year overall survival of patients with NLM on supportive care alone is reported to range between 0% and 46% [5, 7, 11, 17, 18] and the 5-year disease-free survival rate only at 24% [1]. Improvements in patient selection and evaluation, as well as surgical technique and postoperative care, have had a significant effect on this disease and its outcomes. As the majority of these transplantations occurred after 2002, our analysis shows that we are transplanting more patients with better outcomes over time. Olausson et al. [19] and Le Treut et al. [2] have shown that increased selectivity may be too specific, leaving out a number of patients who have already exhausted all other treatment options and who could otherwise benefit from this life-saving therapy. Olausson et al. transplanted 10 patients with expanded criteria, including a higher proliferation rate (measured by Ki67), large tumor burden, and higher age, and were still able to show a 90% 5-year survival [19]. Le Treut et al. developed a selection tool based on the patient's primary tumor location and liver size (not tumor burden), which would have selected 70% of their 85-patient sample to benefit with a 68% five-year overall survival, inappropriately excluding only 2 patients [2]. Thus, the importance of precisely delineating the NET patient best suited for a liver transplantation becomes paramount.

Our analysis allows for greater precision in selecting the ideal NET/NLM patient for liver transplantation. From the inception of the UNOS/OPTN database, our research shows NET patient survival was affected significantly by albumin and total bilirubin, with higher albumin and lower bilirubin being protective. Allograft survival was negatively affected by the need for retransplantation and increased donor creatinine. While the need for retransplantation cannot be selected for, opting for patients with higher albumin levels, lower total bilirubin and donors with lower creatinine can improve both patient and allograft outcomes. While recipient creatinine level has been shown to be a prognostic indicator [3, 20], donor creatinine has not shown the same for liver transplantation recipients at this time. We hypothesize donors with elevated creatinine may have a poorer clinical picture, and thus the donor creatinine may act as a surrogate for the overall state of the liver allograft. Veering away from recipients with higher total bilirubin may help improve allocation efforts toward NET/NLM patients who would better benefit from organ transplantation. Our results corroborate with a large single-center series done by Le Treut et al. [2], finding age as not a significant variable in survival, and this counters a large multivariate analysis done by Lehnert [1]. Along with Le Treut et al., we found that the requirement for early retransplantation was associated with poorer outcomes and serves as a prognostic indicator [2]. However, our analysis found that overall survival of the UNOS/OPTN experience of those transplanted after 2002 is greater than that in their study, 57.8% versus 47%, respectively [2].

Therapeutic approach to liver metastases of NET must consider tumor distribution and bulk. Surgery is generally considered as first-line therapy, specifically liver resection [21]. While liver transplantation may occasionally provide for cure, it more often allows for symptomatic relief and prolongs survival. It is thus reserved for cases in which liver resection is not an option or for recurrent disease. Standard therapies to treat neuroendocrine liver metastases usually fall into the category of liver-directed therapies. This methodology exploits the dual blood supply to the liver, from the hepatic artery and portal vein, in order to control disease. Because of the higher recurrence rate of NET, these techniques are better considered debulking modalities than curative therapy [22]. General guidelines to dictate treatment options are dependent on tumor load, including location and number, as well as size and invasiveness [13]. For fewer lesions, local resection or thermal ablation is recommended. For higher tumor loads or recurrent disease, hepatic artery embolization and chemoembolization or radioembolization is used [23]. Other therapies are nonsurgical, non-liver directed therapies which includes chemotherapy and biotherapy and newer technologies directed at growth factors and peptide receptors, as well as regulation of micro-RNA pathways.

A weakness of this analysis is the inherent nature of the UNOS/OPTN database, as it focuses on clinical data pertinent to liver transplantation and not necessarily the disease process of the liver. The location of the primary NET and the histopathology have been shown to influence overall survival [4, 11], along with concomitant upper abdominal exenteration and presence of hepatomegaly [2]; however, these data points were not included in the UNOS/OPTN database as this database focuses on clinical variables as it pertains to liver transplantation, not necessarily the disease process leading to end-stage liver disease. Additionally, time of diagnosis, presentation of symptoms, production of hormones, and prior treatments utilized were also not available, though quite pertinent in describing this patient population. There is also evidence to suggest Ki67, and E-Cadherin status affects prognosis of NET patients [3, 10, 14, 19], but immunohistochemistry was not recorded. Lastly, recurrence rate and disease-free survivals would allow a more in-depth assessment of outcomes. These weaknesses are acknowledged, and, while whole generalizations cannot be made, it is important to note the breadth of this sample as it illustrates the transplant experience of a national, and thus larger, group of patients with the same rare disease, improving the power of the study. It is important to recognize that liver transplantation for metastatic neuroendocrine tumors is reserved for those with unresectable or refractory disease, both implying a usually dismal prognosis without further treatments. Survival time with patient status is present in all 184 patients of our sample and thus serves as reliable information on the survival experience of this rare and desperate patient population.

While OLT offers a potential for cure in these patients, long-term survival remains lower than the 5-year survival of patients transplanted for other diseases, both malignant and nonmalignant. Many have questioned whether the allocation of limited resources is warranted. Transplanting patients with malignancies has been argued to be justifiable only if the survival can be estimated to exceed 50% at 5 years [24]. Our analysis reveals that not only do NET/NLM patients meet this criteria and do relatively well after transplant but also they continue to improve over time and have survivals not significantly different from HCC patients who are transplanted. Considering the dismal prospects of a patient population that is otherwise without much hope of long-term survival and cure, the impact of these outcomes is compelling [5]. There is promise in the progress of our care for the NLM patient and liver transplantation, which offers us potential for improvement.

## 5. Conclusion

Understanding the limitations of the UNOS/OPTN database and its focus on transplantations and the associated patient and allograft outcomes, this analysis provides valuable insight into patients with NLM and overall survivals after liver transplantation.

While the 5-year survival of patients after OLT for NLM is lower than that after OLT for non-malignant diseases, we argue that the overall survival remains reasonable, exceeding other estimations reported and is not significantly different from HCC outcomes. NET/NLM outcomes after OLT surpass that of patients with untreated NLM left to its natural progression. The significant improvement in outcomes after the introduction of the MELD/PELD scoring system reinforces the potential for gains in transplanting this patient population, who have already failed or exhausted the litany of therapeutic options dedicated to this disease process and who are otherwise facing fatal prospects. This data helps to characterize the NET/NLM patients who have benefited most from liver transplantation. Stratification of variables show requirement of a retransplant; decreased albumin level of the recipient and elevated donor creatinine influence the prognosis of the patient and the allograft after OLT in patients with NLM. In this era of transplantation, we argue the outcomes of liver transplantation for a carefully chosen subset of neuroendocrine tumor patients are acceptable and potentially life-saving.

## Figures and Tables

**Table 1:** Clinical Variables used for univariate analysis with significance for patient and allograft survival. *P* values ≤ 0.2 were considered significant for multivariate analysis and marked in bold for clarity.

Univariate Cox regression		
Clinical variables	Significance for patient survival	Significance for allograft survival

*Recipient variables:*		
Age	.477	.454
Gender	**.168**	**.157**
Education level	**.174**	.304
Ethnicity	**.007**	**.024**
Body mass index	**.083**	**.043**
MELD/PELD	**.000**	**.006**
Days on waitlist	**.017**	**.033**
Previously transplanted	.782	**.079**
Patient requires retransplantation	.394	**.000**
Dialysis 1 week prior to OLT	**.006**	**.003**
Encephalopathy	**.052**	**.135**
Ascites	**.051**	**.088**
Multiorgan recipient	**.110**	**.095**
Type of allograft (Whole or Split)	.244	**.118**
Creatinine at OLT	**.021**	**.082**
Total bilirubin at OLT	**.008**	**.013**
INR at OLT	.546	.796
Albumin at OLT	**.000**	**.001**
Cold ischemia time	.574	.335
Warm ischemia time	.650	.692
Length of postoperative stay	**.001**	**.002**

*Donor variables:*		
Age of donor	**.097**	**.037**
Creatinine of donor	**.015**	**.067**
Total bilirubin of donor	**.099**	**.095**
AST of donor	.639	.882
ALT of donor	.447	.753

**Table 2:** Multivariate Cox regression results of clinical variables found to be significant (*P* ≤ 0.05). *Signifies percentage of sample who required retransplantation.

	Range	Mean	*P* value	Change in OR
*Transplants after 1988*				
Patient survival				
Albumin at transplant	0.9–3.2	1.166	0.033	0.446
Allograft survival				
Patient required retransplant	n/a	7.6%*	<0.00	49.02
Donor creatinine	0.30–15.0	1.38	0.004	1.32

*Transplants after 2002*				
Patient survival				
Total bilirubin at transplant	0.10–69.60	2.64	0.02	1.063
Albumin at transplant	1.40–5.20	3.83	0.011	0.480
Donor creatinine	0.3–15.0	1.46	0.004	1.288
Allograft survival				
Patient required retransplant	n/a	4.5%*	<0.00	35.89
Total bilirubin at transplant	0.10–69.60	2.64	0.019	1.060
Albumin at transplant	1.40–5.20	3.83	0.006	0.455
Donor creatinine	0.3–15.0	1.46	0.004	1.308

## References

[1] Lehnert T (1998). Liver transplantation for metastatic neuroendocrine carcinoma: an analysis of 103 patients. *Transplantation*.

[2] Le Treut YP, Grégoire E, Belghiti J (2008). Predictors of long-term survival after liver transplantation for metastatic endocrine tumors: an 85-case French multicentric report. *American Journal of Transplantation*.

[3] Sharma P, Schaubel DE, Guidinger MK, Merion RM (2009). Effect of pretransplant serum creatinine on the survival benefit of liver transplantation. *Liver Transplantation*.

[4] Steinmüller T, Kianmanesh R, Falconi M (2008). Consensus guidelines for the management of patients with liver metastases from digestive (neuro)endocrine tumors: foregut, midgut, hindgut, and unknown primary. *Neuroendocrinology*.

[5] Frilling A, Sotiropoulos GC, Li J, Kornasiewicz O, Plöckinger U (2010). Multimodal management of neuroendocrine liver metastases. *Journal of the International Hepato Pancreato Biliary Association*.

[6] http://optn.transplant.hrsa.gov/..

[7] Mayo SC, De Jong MC, Bloomston M (2011). Surgery versus intra-arterial therapy for neuroendocrine liver metastasis: a multicenter international analysis. *Annals of Surgical Oncology*.

[8] Modlin IM, Pavel M, Kidd M, Gustafsson BI (2009). Review article: somatostatin analogs in the treatment of gastro-entero-pancreatic neuroendocrine (carcinoid) tumors. *Alimentary Pharmacology & Therapeutics*.

[9] Coppa J, Pulvirenti A, Schiavo M (2001). Resection versus transplantation for liver metastases from neuroendocrine tumors. *Transplantation Proceedings*.

[10] Mazzaferro V, Pulvirenti A, Coppa J (2007). Neuroendocrine tumors metastatic to the liver: how to select patients for liver transplantation?. *Journal of Hepatology*.

[11] van Vilsteren FGI, Baskin-Bey ES, Nagorney DM (2006). Liver transplantation for gastroenteropancreatic neuroendocrine cancers: defining selection criteria to improve survival. *Liver Transplantation*.

[12] Glazer ES, Tseng JF, Al-Refaie W (2010). Long-term survival after surgical management of neuroendocrine hepatic metastases. *Journal of the International Hepato Pancreato Biliary Association*.

[13] Touzios JG, Kiely JM, Pitt SC (2005). Neuroendocrine hepatic metastases: does aggressive management improve survival?. *Annals of Surgery*.

[14] Grossman EJ, Millis JM (2010). Liver transplantation for non-hepatocellular carcinoma malignancy: indications, limitations, and analysis of the current literature. *Liver Transplantation*.

[15] http://www.unos.org/..

[16] Punch J, Gish RG (2006). Model for end-stage liver disease (MELD) exception for uncommon hepatic tumors. *Liver Transplantation*.

[17] Madoff DC, Gupta S, Ahrar K, Murthy R, Yao JC (2006). Update on the management of neuroendocrine hepatic metastases. *Journal of Vascular and Interventional Radiology*.

[18] Rothenstein J, Cleary SP, Pond GR (2008). Neuroendocrine tumors of the gastrointestinal tract: a decade of experience at the princess margaret hospital. *American Journal of Clinical Oncology*.

[19] Olausson M, Friman S, Herienius G (2007). Orthotopic liver of multivisceral transplantation as treatment of metastatic neuroendocrine tumors. *Liver Transplantation*.

[20] Xu N, Yan LN, Yang JY (2011). New prognostic model for adult-to-adult living donor liver transplant recipients. *Transplantation Proceedings*.

[21] Blonski WC, Reddy KR, Shaked A, Siegelman E, Metz DC (2005). Liver transplantation for metastatic neuroendocrine tumor: a case report and review of the literature. *World Journal of Gastroenterology*.

[22] Harring TR, Nguyen NTT, Goss JA (2011). Treatment of liver metastases in patients with neuroendocrine tumors: a comprehensive review. *International Journal of Hepatology*.

[23] Ahlman H, Friman S, Cahlin C (2004). Liver transplantation for treatment of metastatic neuroendocrine tumors. *Annals of the New York Academy of Sciences*.

[24] Touzios JG, Krzywda B, Nakeeb A, Pitt HA (2005). Exercise-induced cholangitis and pancreatitis. *Journal of the International Hepato Pancreato Biliary Association*.

